# Effectiveness of Gastric Cancer Endoscopic Screening in Intermediate-Risk Countries: Protocol for a Systematic Review and Meta-Analysis

**DOI:** 10.2196/56791

**Published:** 2025-02-03

**Authors:** Maria Beatriz Mourato, Nuno Pratas, Andreia Branco Pereira, Filipa Taré, Raphael Chança, Inês Fronteira, Rui Dinis, Miguel Areia

**Affiliations:** 1 NOVA National School of Public Health, Public Health Research Centre, Comprehensive Health Research Center, CHRC, LA-REAL, CCAL, NOVA University Lisbon, Lisbon, Portugal Lisbon Portugal; 2 Unidade Local de Saúde do Alto Alentejo Hospital Doutor José Maria Grande Portalegre Portugal; 3 Divisão de Avaliação de Tecnologias em Saúde Rio de Janeiro Brazil; 4 Instituto Nacional do Câncer Rio de Janeiro Brazil; 5 Hospital do Espírito Santo de Évora Évora Portugal; 6 Instituto Português de Oncologia de Coimbra Coimbra Portugal

**Keywords:** gastric cancers, endoscopic screening, intermediate-risk countries, neoplasia, early detection, diagnosis, cancer screening, survival, meta-analysis, gastrointestinal cancers

## Abstract

**Background:**

Gastric cancer (GC) is the fifth most prevalent neoplasm worldwide and the fourth with the highest mortality, and its geographical distribution is not homogeneous with high-risk, intermediate-risk (IR), and low-risk areas. Advanced stages at diagnosis are related to high mortality, but early detection greatly increases the chances of survival. Upper endoscopy with biopsy is the gold standard for GC diagnosis. Several studies have investigated the relevance of endoscopic screening and how to implemente it in IR countries. However, most Western societies recommend screening only in selected populations with high-risk factors for GC. No systematic reviews on GC endoscopic screening in IR countries exist.

**Objective:**

We aimed to determine the effectiveness of endoscopic GC screening in IR countries.

**Methods:**

We will include randomized and nonrandomized controlled trials, cohort studies, case-control studies, cross-sectional studies, and economic studies focusing on endoscopic screening of GC in the asymptomatic population of IR countries. The search will be conducted in MEDLINE, SCOPUS, Embase, and Web of Science. Other gray literature sources will be additionally searched. Studies published in English, Portuguese, or Spanish until September 2024 will be included. Two independent reviewers will screen the titles and abstracts of all search results. The selected studies will then be fully analyzed, and the data will be collected and coded in a database. To minimize the risk of bias, the included studies will undergo a quality analysis according to Cochrane risk of bias tools, RoB 2 of randomized trials and ROBINS-I for nonrandomized trials; Newcastle-Ottawa Quality Assessment Scale for case-control and cohort studies; and National Heart, Lung and Blood Institute study quality assessment tools for cross-sectional studies. The data collected will be cataloged in 2 categories: efficacy or effectiveness data and economic data, and separate meta-analyses will be performed for each category if appropriate.

**Results:**

This study is expected to provide results on the efficacy, effectiveness, and cost-effectiveness of endoscopic screening in an IR population. To date, 969 studies were screened for title and abstract, 75 were selected for full-text screening, and 44 were retained for data analysis. Additionally, 2 studies were selected from our manual search. Currently, the study is in the early stages of data extraction and risk of bias assessment and is expected to be published in the first quarter of 2025.

**Conclusions:**

To our knowledge, this review will be the first to provide evidence on the effectiveness of endoscopic GC screening in IR countries. In doing so, we believe we will help guide future research, inform health care decisions and assist policy makers in this area, and support future decisions to implement GC screening programs in this type of population.

**Trial Registration:**

PROSPERO CRD42024502174; https://www.crd.york.ac.uk/prospero/display_record.php?RecordID=502174

**International Registered Report Identifier (IRRID):**

DERR1-10.2196/56791

## Introduction

Gastric adenocarcinoma, also known as gastric cancer (GC), is a malignant neoplasm resulting from anarchic growth of gastric mucosal gland cells [1]. It is a heterogeneous disease with multiple clinical, histological, and molecular variables that influence its presentation [1,2].

According to data from The Global Cancer Observatory 2022, GC is the fifth most common and the fifth most lethal neoplasm worldwide [3]. Its geographical distribution is not homogeneous: there are high-risk areas with incidence 20 and more (age-standardized rate measured per 100,000 people-year; eg, Japan, South Korea, or Mongolia); intermediate-risk (IR) areas with age-standardized rate 10 and more and less than 20 (eg, Portugal or China); and low-risk areas with age-standardized rate 10 or less (United States, United Kingdom, Switzerland, or Germany) [2,3].

Diagnosis at an advanced stage and the aggressiveness of the disease result in a 5-year survival rate of between 20% and 40% [4]. In contrast, early-stage GC has an excellent prognosis, with a 5-year survival rate of greater than 90%, and can often be treated with minimally invasive and organ-sparing techniques such as endoscopic resection [5]. Therefore, early detection of cancer greatly increases the chances of successful treatment. The 2 components of cancer screening are early diagnosis and screening. The former focuses on detecting symptomatic patients as early as possible, while the latter involves testing healthy individuals to identify those with the disease before symptoms occur [6]. The symptoms of stomach cancer are nonspecific and usually develop late, meaning that detection based on symptoms would not detect the disease in its early stages. On the other hand, screening programs should be implemented when: their effectiveness has been demonstrated; the resources needed to implement them are sufficient to cover the target population; there are facilities to confirm the diagnosis, treatment, and follow-up of those with positive results; and the prevalence of the disease is high enough to justify the effort and cost of the screening program [6]. Therefore, the decision to implement the screening program requires that these conditions are met. Several methods have been proposed to screen for GC, namely serological markers, biomarkers, molecular or genetic tests, or more invasive techniques such as upper gastrointestinal endoscopy [7]. The latter, which allows direct visualization of the gastric mucosa and collection of biopsies for histological examination, is considered the gold standard technique for definitive diagnosis of GC [8].

In some high-risk countries, such as Japan and South Korea, GC screening programs have been in place for several years and have been shown to reduce mortality and increase early detection and 5-year survival rates [9-11]. The same has been demonstrated in screening programs developed in China [12,13].

In Western IR countries, several studies have been developed to determine the relevance of screening and how it might be developed. Of particular importance was the study developed by Areia et al [14], which showed that endoscopic screening for GC in IR countries can be cost-effective when combined with endoscopic screening for colorectal cancer. This has been included in the recommendations of the United European Gastroenterology and the European Society of Gastrointestinal Endoscopy [14-16]. Despite this evidence and the recommendations, most Western Societies continue to recommend screening only in selected populations with high-risk factors for GC [16,17]. Furthermore, and to the best of our knowledge, there are no systematic reviews on GC endoscopic screening in IR countries. With this systematic literature review, we aim to analyze the scientific evidence published until September 2024 on the cost-effectiveness of endoscopic screening for GC in IR countries.

This study aims to determine the effectiveness and economic viability of endoscopic GC screening in IR countries**,** which will answer the research question, “What is the effectiveness of endoscopic screening for GC in IR countries?”

These objectives are defined according to the Population, Intervention, Comparison, Outcome and Study Design (PICOS) framework (Textbox 1).

**Population, Intervention, Comparison, Outcome and Study Design (PICOS) framework for the systematic review and meta-analysis.**

**PICOS question: What is the effectiveness of endoscopic screening for gastric cancer (GC) in intermediate-risk countries?**

- Population:
  Asymptomatic population of intermediated-risk countries (countries with incidence age-standardized rate 10-20 per 100.000 person/years: Tajikistan, Iran, Azerbaijan, Kyrgyzstan, Bhutan, Belarus, Peru, Mali, Chile, Costa Rica, Democratic People Republic of Korea, China, Kazakhstan, Russian Federation, Viet Nam, Estonia, Colombia, Portugal, Ecuador, Albania, Guadeloupe [France], Guatemala, Latvia, Armenia, Turkmenistan, Myanmar, Samoa, Turkey, Lithuania, Lao People’s Democratic Republic, Sao Tome and Principe, Afghanistan, Martinique [France], Brunei Darussalam, Zimbabwe, and Uzbekistan), between 40 and 80 years of age, without diagnostic of GC or precancerous lesions.
- Intervention: Endoscopic screening for GC.
- Comparison: No screening for GC.
- Outcome: The effectiveness of endoscopic screening of GC is defined as the detection rate of Helicobacter pylori; detection rate of precancer lesions; detection rate of GC; detection rate of early GC; stage at diagnosis; mortality rate of GC of screened versus nonscreened patients; 5-year survival rate of GC screened; and costs of screening program.
- Study designs: Randomized controlled trials, nonrandomized controlled trials, cohort studies, case-control studies, cross-sectional studies, and cost-effectiveness studies.

## Methods

This study will follow the Preferred Reporting Items for Systematic Reviews and Meta-Analyses (PRISMA) guidelines [18] and PICOS criteria for comprehensive assessment.

### Eligibility Criteria

The inclusion criteria include studies published as free full papers in English, Portuguese, or Spanish until September 2024, from countries with an IR for GC (countries with incidence age-standardized rate 10-20 per 100,000 person/years: Tajikistan, Iran, Azerbaijan, Kyrgyzstan, Bhutan, Belarus, Peru, Mali, Chile, Costa Rica, Democratic People Republic of Korea, China, Kazakhstan, Russian Federation, Viet Nam, Estonia, Colombia, Portugal, Ecuador, Albania, Guadeloupe [France], Guatemala, Latvia, Armenia, Turkmenistan, Myanmar, Samoa, Turkey, Lithuania, Lao People’s Democratic Republic, Sao Tome and Principe, Afghanistan, Martinique [France], Brunei Darussalam, Zimbabwe, Uzbekistan); eligible study designs are randomized controlled trials, nonrandomized controlled trials, cohort studies, case-control studies, cross-sectional studies, and cost-effectiveness studies; and no filters or restrictions related to year of publication or publication status, will be applied.

The exclusion criteria include systematic reviews and other types of reviews, meta-analyses, case series, case reports, and other publication types such as editorials, commentaries, notes, letters, and opinions.

### Information Sources

The information sources for this systematic review are electronic databases (MEDLINE, SCOPUS, Embase, and Web of Science). To capture additional studies (gray literature), manual searches will include published abstracts from the most relevant international gastroenterology and endoscopy conferences, clinical trial registries for ongoing studies, reference lists of included studies or other published reviews or meta-analyses. Authors of unpublished studies or published studies in which data are missing will be contacted to confirm eligibility.

### Search Strategy

The search strategy follows the Peer Review of Electronic Search Strategies (PRESS) guidelines [19]. The search will be conducted in MEDLINE, SCOPUS, Embase, and Web of Science and the search strategy will be tailored to each database using database-specific search terms (Multimedia Appendix 1).

### Selection Process

The references, including the abstract of studies retrieved after searching each database, will be imported to Rayyan (Rayyan Systems, Inc), an open-source software that allows several reviewers to blindly access the inclusion or exclusion of studies in literature reviews registering the entire process. This software will analyze and merge the potential duplicates, under operator validation. The initial selection process will be carried out by 2 independent reviewers (MBM and NP) based on the title and abstract. Studies will be classified as “included,” “excluded,” or “maybe.” The selection process will be blinded and supervised by 2 other independent authors (FT and ABP). Conflicts and “maybe” assessments will be resolved between the 2 reviewers with the support of 2 authors (FT and ABP). After the final list of included studies is obtained, the full texts are retrieved. The full text of each study will be analyzed by 3 independent reviewers (MBM, NP, and ABP), and a decision taken on inclusion or exclusion. The list of items included is exported to a Microsoft Excel database where the data to be analyzed are entered.

The agreement rate will be calculated using Cohen κ, Egger regression, and Begg regression and will be reported at all stages of the selection process (title screening, abstract screening, and full-text screening).

### Data Collection Process

Data from included studies will be collected by 2 reviewers (MBM and NP) and inserted into a Microsoft Excel database with data coding. The data collected will be cataloged in 2 categories: efficacy or effectiveness data and economic data, and separate meta-analyses will be performed for each of these categories.

For the statistical analyses and meta-analysis of the data collected, the authors will use SPSS (version 29.0.2.0; IBM Corp) and Jamovi (version 2.5; The jamovi Project). Bibliographic references will be managed in Zotero (Corporation for Digital Scholarship) software. Artificial intelligence tools may be used to extract and analyze data.

### Data Items

The data collection form will include the items or variables defined in outcomes (Table 1) and other variables (Table 2).

**Table 1:** Variables related to outcomes of screening and cost-efficiency.

Variable	Definition or domain
Endoscopic screening	Number of individuals screened by endoscopy.
Frequency of screening	Frequency of endoscopic screening in years.
Age range covered by screening	Age at start and end of screening.
Screening adherence rate	Percentage of invited individuals that did the screening.
Number of biopsies	Total number of biopsies performed in the study.
*Helicobacter pylori* diagnosis rate	Percentage of individual screened that were diagnosed with *Helicobacter pylori*.
Detection rate of premalignant lesions	Number of premalignant lesions (atrophic gastritis, intestinal metaplasia and low-grade intraepithelial neoplasia, formerly low-grade dysplasia) / total number of upper endoscopies performed.
Gastric cancer detection rate	Number of gastric cancers diagnosed by screening endoscopy / total number of screening upper endoscopies performed.
Early gastric cancer detection rate	Number of early gastric cancers (high-grade intraepithelial neoplasia and mucosal adenocarcinoma) detected by endoscopic screening / total number of gastric cancers detected by endoscopic screening.
Lethality rate	Number of people who died from gastric cancer diagnosed by endoscopic screening / population at risk during the study period.
5-year survival rate in patients diagnosed with gastric cancer at screening	Percentage of patients diagnosed with stomach cancer at screening who live at least 5 years after diagnosis.
Incremental cost-effectiveness ratio	Value (in euro) of implementing the upper endoscopy screening program (or adding it to other existing screening programs, eg, endoscopic screening for colorectal cancer).

**Table 2:** Other variables

Variable	Domain
Study Bibliographic Reference	Reference
Country or countries of origin of the study	Country
Study design	Randomized controlled trial Nonrandomized controlled trial Cohort Case-control Cross-sectional Cost-effectiveness
Population or sample	Base population potentially to be screened by upper endoscopy
Participants	Age Sex distribution

### Risk of Bias in Individual Studies

To minimize the risk of bias, the included studies will undergo a quality analysis according to Cochrane risk of bias tools, RoB 2 of randomized trials and ROBINS-I, for nonrandomized trials; Newcastle-Ottawa Quality Assessment Scale [20] for case-control and cohort studies; and National Heart, Lung and Blood Institute study quality assessment tools [21] for cross-sectional studies. The Consensus on Health Economic Criteria (CHEC) list [22] will be used for the assessment of the methodological quality of cost-effectiveness studies.

Studies with low quality or high risk of bias will be reported and not be used for meta-analysis.

### Data Synthesis

If it is possible to collect quantitative data from the selected studies, we will perform 2 separate meta-analysis: one to report the effect size of 5 outcomes in studies reporting efficacy or effectiveness of endoscopic screening for GC (detection rate of premalignant lesions, detection rate of GC, detection rate of early GC, and 5-year survival rate and mortality of GC diagnosed in screening programs); other to report the effect size of cost-effectiveness of endoscopic screening for GC in IR countries.

If appropriate (at least 2 studies per outcome) [23], pooled rates and odds ratio along with 95% CIs will be calculated for GC detection, early GC detection, adherence to the screening program, and GC mortality using random-effects model, using SPSS, Jamovi, and Meta-Essentials for Microsoft Excel (Erasmus Research Institute of Management). The *I*^2^ homogeneity will also be performed to check the need for subgroup analysis (moderation analysis).

If it is not possible to carry out a meta-analysis due to insufficient data, an executive summary is prepared that summarizes the data from the studies included in the systematic review.

### Meta-Bias(es)

After evaluating the biases of individual studies and ensuring that all studies that may be of interest for their conclusions are integrated into the review, a summary of the risks of bias in the meta-analysis or meta-synthesis will be presented.

### Confidence in Cumulative Evidence

The strength of the body of evidence will be assessed with Grading of Recommendations, Assessment, Development and Evaluation (GRADE) [24].

## Results

**Figure 1:**
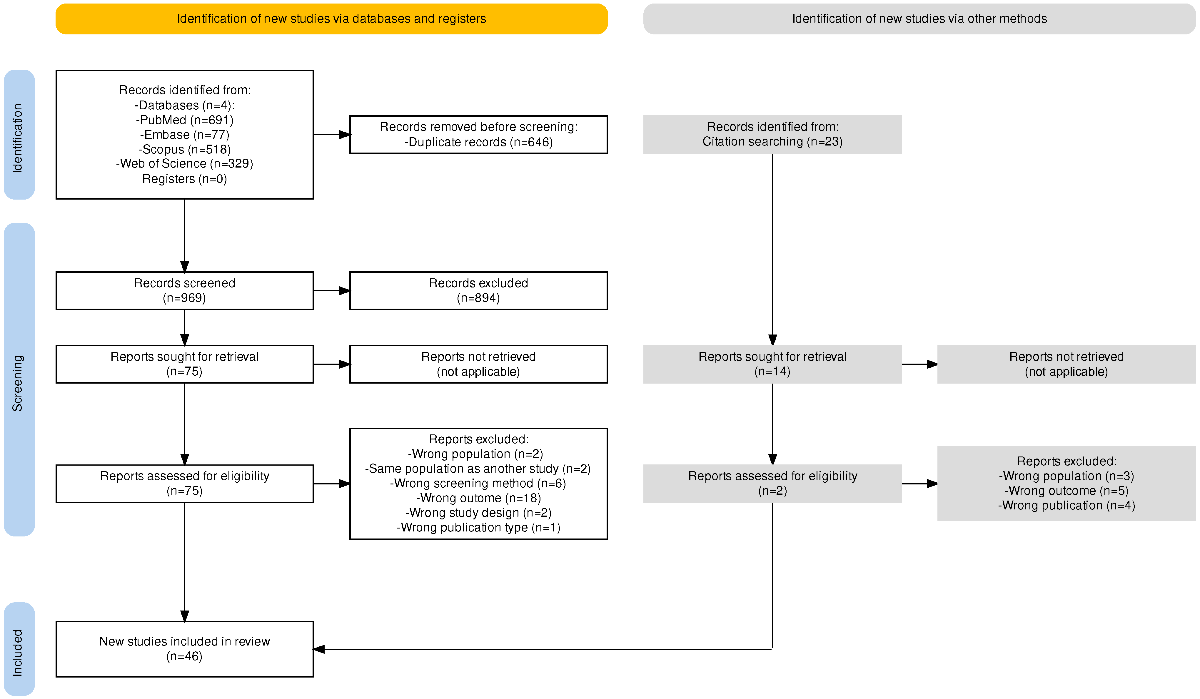
Flowchart of PRISMA (Preferred Reporting Items for Systematic Reviews and Meta-Analyses). NA: not applicable.

Our initial search of the 4 electronic databases, using descriptors adapted for each database, identified 1615 studies of potential interest (Figure 1). After excluding duplicates, 969 studies were screened for title and abstract. Of these, 75 were selected for full-text screening. We retained 44 studies for data analysis and the remaining 31 studies were excluded for the following reasons: wrong population (n=2), same population as another study (n=2), wrong screening method (n=6), wrong outcome (n=18), wrong study design (n=2), and wrong publication type (n=1). In addition, our manual search identified 23 publications of potential interest, of which 2 were selected for the data extraction phase. Currently, the study is in the early stages of data extraction and risk of bias assessment and is expected to be published in the first quarter of 2025.

## Discussion

This protocol outlines the methods for a systematic literature review and meta-analysis of published primary scientific studies on the effectiveness of endoscopic GC screening in IR countries.

This study will provide results on the following outcomes: frequency of screening, age range covered by screening, screening adherence rate, number of biopsies, *Helicobacter pylori* diagnosis rate, detection rate of premalignant lesions, GC detection rate, early GC detection rate, lethality rate, 5-year survival rate in patients diagnosed with GC at screening, and incremental cost-effectiveness ratio.

To the best of our knowledge, this is the first systematic review of the effectiveness of endoscopic screening for GC in IR countries and it is expected that the presentation of these results will shed light on the relevance of endoscopic GC screening in these populations. The benefit of performing screening upper endoscopy for asymptomatic individuals for GC remains controversial [25]. Due to the high burden of GC, countries in East Asia such as Japan and Korea have implemented nationwide population-based GC screening strategies to reduce incidence and mortality [9,26]. Other studies have shown that endoscopic screening for GC is cost-effective in the IR to high-risk population [14,27]. However, to date, we have not found a systematic review that synthesizes the results of all studies conducted in IR countries, and for this reason, our systematic review is of particular interest to inform health policy makers in IR countries about the effectiveness of endoscopic screening for GC in this type of population.

An extensive search of databases and gray literature will be conducted to identify all relevant studies. However, this review may have some important limitations. First, it is important to bear in mind that the majority of IR countries, especially Western countries, do not yet have population-based screening for this pathology, which may make it difficult to obtain primary studies on this topic from these countries. Second, although China is an IR country with endoscopic screening for GC, some studies reporting the results of these screening programs are written in Chinese and cannot be included in our review. Nevertheless, we will conduct an intensive search of the databases to ensure that we obtain studies written in English, Portuguese, or Spanish that report the results of these screening programs. Third, on the other hand, the Chinese territory is so large that risk varies greatly between different areas of the country, which may introduce a significant bias if some included studies are based only on results of high-risk areas, as this type of population may have different characteristics from those observed in other areas of China or other IR countries. These limitations will be reported in the final paper of the systematic review so that our results can be interpreted rigorously and truthfully. The final paper of the systematic review and meta-analysis will be published in the first quarter of 2025.

With this systematic review and meta-analysis, we hope to contribute to the design of GC screening strategies in IR countries, with the primary goal of reducing mortality from this disease. In the future, it may be necessary to update this systematic review as a new consortium. Towards Gastric Cancer Screening Implementation in the European Union [28] has recently been established in Europe to study and implement GC screening in this area.

## Appendix

Multimedia Appendix 1 Search strategy.

## Abbreviations

CHEC: Consensus on Health Economic Criteria
GC: gastric cancer
GRADE: Grading of Recommendations, Assessment, Development and Evaluation
IR: intermediate-risk
PICOS: Population, Intervention, Comparison, Outcome and Study Design
PRISMA: Preferred Reporting Items for Systematic Reviews and Meta-Analyses
PRESS: Peer Review of Electronic Search Strategies

## Data Availability

All data generated or analyzed during this study are included in this published article (and its supplementary information files).
